# Particulated wisdom teeth as an autologous bone substitute for grafting/filling material in bone defects: Case Report

**DOI:** 10.4317/jced.56547

**Published:** 2020-04-01

**Authors:** Ivan Arabadzhiev, Peter Maurer, Eber Stevao

**Affiliations:** 1 Master of Dental Medicine; Master of Public Healthcare and Healthcare Management; Resident in Oral Surgery at Praxisklinik Prof. Dr. Dr. Peter Maurer in Sankt Wendel - Saarland, Germany; 2 Medical Doctor; Dental Doctor; OMF surgeon at Praxisklinik Prof. Dr. Dr. Maurer in Sankt Wendel - Saarland, Germany; 3 Doctor of Dental Surgery, OMF surgeon, PhD in OMFS, PostDoctoral at Baylor University Medical Center and Baylor College of Dentistry, Dallas - TX, USA, Senior OMF Consultant surgeon at Stevao & Sons Consulting Limited

## Abstract

**Introduction:**

Bone augmentation material using permanent teeth are confirmed in many articles to provide good histological and clinical results. Advances in osteoconduction and osteoinduction, low cost of material, no risk of disease transmission and elimination of foreign body reaction are important aspects not only to the clinician but to patient as well. Many techniques and devices to obtain graft material for bone augmentation out of teeth are found in the literature.

**Material and Methods:**

The wisdom teeth crush technique developed in our office and described in this case report is simple and no specific devices for teeth grinding were used. The graft material was obtained from impacted intact wisdom teeth without chemical conditioning of the particles with preservation of the dental pulp and the cells found in it. This study presents crushed autologous maxillary wisdom tooth as filling material in two bone defects in premaxilla caused by cysts removal.

**Results:**

The clinical results and Panoramic X-ray evaluation at three months postoperatively were very promising.

**Conclusions:**

Although further clinical researches are necessary to evaluate this substitutive for bone augmentation technique, the authors believe it can be safely used by oral/maxillofacial surgeons.

** Key words:**Particulated wisdom tooth, autologous augmentation, bone substitutes, wisdom teeth crush technique.

## Introduction

The autogenous bone is considered to be the gold standard for bone augmentation due to its osteoconductive, osteoinductive and osteogenic properties (1).

The quantity and size of donor bone, the additional trauma, the postoperative graft resorption and prolonged operation time are limiting factors for its use. There is great number of substituting materials which are available on the market nowadays and they can be classified as: a) Allograft, b) Xenograft, and c) Alloplastic graft.

Based on the potentials of osteoconduction, osteoinduction, and osteogenesis through growth factors present in a tooth and similar histogenesis between tooth and bone, the inorganic and organic components of an extracted tooth should be considered as graft material (2).

Accordingly with Kim *et al.* (2), about 90% of dentinal organic components are known to be type I collagens. The rest of organic components are non collagen proteins that contribute to mineralization - osteocalcin, osteonectin, phosphophoryn, dentin sialoprotein, dentin-specific extracellular matrix protein. Osteopontin is known to trigger osteogenesis through the early differentiation of the osteoblasts and is manifested after grafting the tooth graft material on alveolar bone defects in rats.

The osteoinductivity of bone morphogenetic protein (BMP) extracted from animal teeth has been confirmed. Dentin and cementum contain various other growth such as insulin-like growth factor (IGF), platelet-derived growth factor (PDGF), fibroblast growth factor (FGF), and transforming growth factor (TGF)-β (2).

BMP extracted from human dentin matrix induced new bone formation into muscle pouches in rats. Dentin-matrix-derived BMP is probably not identical to, but is similar to, bone-matrix-derived BMP, though both types of BMP have the same action *in vivo* (3).

It is well known that dentoalveolar ankylosis results in osseous replacement and formation of a new bone. This principle is used for preservation of the height and width of the alveolar bone crest after trauma. Another fact is that dentin promotes new bone formation when located close to native cortical bone and a bone graft material composed of milled tooth promotes early healing and bone formation (1).

-Bibliographic review

Autogenous demineralized dentin graft has been developed and implemented in clinical practice for more than 10 years (4).

Many different ways for preparation of the extracted teeth into a bone augmentation material can be found in the literature: 1) Demineralized dentin 4, 2) Freeze Dried Dentin, 3) Grinding with special dentin grinder and chemical disinfection (5,6), 4) Grinding with domestic Grinder (7), 5) Grinding with conventional bone mill and no chemical conditioning (8), 6) Just crushing without any chemical or thermal preparation (9-11).

Nampo *et al.* compared (9) iliac bone and particulate teeth augmented defects in rat jaws. The jawbone formation was measured using real-time polymerase chain reaction, microcomputed tomography, and histologic analysis. Polymerase chain reaction, hematoxylin- eosin staining and microcomputed tomography showed that after eight weeks, tooth graft material produced a similar amount of new bone compared to iliac bone graft material. 

A special grinding device (dentin grinder) is able to process extracted teeth simultaneously after the extraction into grafting material. With this process the particles need to be additionally conditioned/disinfected with chemicals following special protocol, so the new dentin particles could be inserted in the extraction socket.

Calvo-Guirado *et al.* (5) concluded that in this way bone grafts could be considered to be an attractive option due to their autogenous origin and favorable clinical results, which have shown that these materials offered good osteoinductive capacities. The particulate tooth material provides excellent biocompatibility without eliciting an immune response or a foreign material reaction, or infection after it is used. In addition, it has osteoinduction, osteoconduction, and progressive substitution capabilities, and it can be processed to form various sizes and shapes.

Binderman *et al.* (6) reported that autogenous mineralized dentin particulate that is grafted immediately after extractions should be considered as the gold standard for socket preservation, bone augmentation in sinuses or filling bone defects. 

Different simplified techniques were presented in the literature as preparation protocol for autogenous teeth graft.

Valdec *et al.* (8) used conventional bone mill to grind extracted teeth after removal of pulp, enamel and cement. Without chemical or physical disinfection the material was inserted in the alveoli. No clinical signs of significant infection or graft loss were present. It was shown that particulated dentin of autologous teeth may serve as an alternative to autologous bone for alveolar ridge preservation.

An article presented by Joshi *et al.* (7) whole caries-free teeth were grinded using a conventional domestic grinder. The particles after being conditioned with lactic acid for disinfection and partial demineralization were inserted into sockets. The control group were augmented with β-TCP and ungrafted sites. In the process of collecting bone biopsy, teeth grafted sites were found to be have comparatively harder consistency than β-TCP-grafted sites. Histologically teeth grafted sites showed newly formed bone associated with connective tissue stroma rich in angiogenesis. Teeth derived graft has shown more promising results as compared to β-TCP in achieving minimal volumetric alveolar bone loss allowing predictable, consistent, and reproducible bone regeneration.

The grafting protocol clinically tested and published by Pohl *et al.* (10) and (11) did not use any chemicals or special grinding devices. Fully impacted teeth were crushed into particles. The graft material contained dentin, enamel and cementum fragment. The mix was used for Sinus-Lift and restoration of lateral and intraosseous defects of the alveolar ridge. The histological examination showed osteoconductive osteogenesis with encapsulation of tooth enamel and dentin portions and partial resorption of the tooth components. Cementum shares were no longer discernible. The immunohistochemical assessment showed intense new vessel formation. The use of autogenous crushed tooth material from impacted third molars may represent an alternative augmentation material.

It is known micro-grafts derived from dental pulp are a useful method for bone regeneration.5

The dental pulp is able to form mineralized matrices that do not always resemble dentin accordingly with Hosoya *et al.* (12) in his study where dental pulp was transplanted into subcutaneous tissue in rats. Seven days after transplantation, initial hard tissue was formed. After fourteen days this hard tissue expanded. The mineralized matrix was immunopositive for osteocalcin, osteopontin, and bone sialoprotein, indicating that pulp cells possessed the ability to form a bone- or cementum-like matrix. Calcification of the matrix may occur in necrotic cells and matrix vesicles, followed by collagenous calcification.

Dental pulp cells produced bone instead of dentin when those cells were implanted with HA/TCP powder as their carrier. This evidence shows that dental pulp cells are the common progenitors of odontoblasts and osteoblasts, or dental pulp cells are mesenchymal stem cells themselves (13).

*In vitro* dental pulp stem cells have extensive differentiation ability and the demonstrated interactivity with biomaterials makes them ideal for tissue reconstruction. These cells are multipotent stromal ones that can extensively proliferate, have a long lifespan and build *in vivo* an adult bone with Haver’s channels and an appropriate vascularization (14).

## Case Report

A 17- year old patient was referred to our Oral and Maxillofacial Surgery office because of a periapical cysts involving teeth 12 and 22 (FDI World Dental Federation notation) and small periapical radiolucencies involving teeth 13 and 23.

On clinical examination no carious lesions were present and the patient never experienced any pain in the past. These teeth were not periodontically compromised. Vitality test with cold-spray and hit showed absence of reaction on teeth 12 and 22, although subnormal pathological reaction on teeth 13 and 23. It lead the examiners to conclude that devitalization of all four teeth was present and the cyst formation on the anterior premaxilla was related with repetitive chronic trauma to patient´s upper frontal teeth due to martial arts practice in the past without proper dentition protection.

**Figure 1:**
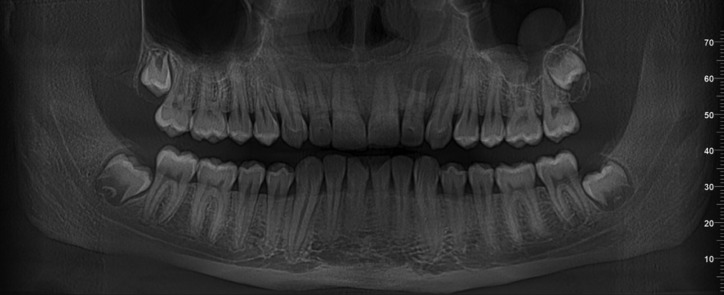
Preoperative panoramic view X-ray reconstruction from a CBCT. It is possible to observe the size of two cystic lesions involving teeth 12 and 22. Also, four impacted third molars present and an incidental left maxillary sinus mucosal cyst finding.

The Cone Beam Computed Tomography (CBCT) imaging showed a cystic lesion involving tooth 22, measuring 8.3 x 5.4 mm and tooth 22, measuring 13.5 x 10.7 mm (Figure 1). After discussion with patient and his parents, based on all therapeutic options and his best interest, the treatment chosen was root canal treatment, apicoectomy of teeth number 13, 12, 22, 23, cystectomy of both cysts, and bone substitute augmentation/filling in the area with autologous graft material obtained from wisdom teeth particulation. Teeth number 18 and 28 (dystopically) impacted with tooth calcification Nolla´s15 stage 7 for tooth number 18 and stage 6 for tooth number 28, as they can be seen on Figure 1.

Prior to surgical intervention endodontic treatment was performed on teeth 13, 12, 22 and 23 in two sessions under local anesthesia. At the first visit teeth had their dental canals accessed with proper palatal endodontic cavities for hand instrumentation. During the treatment there was no bleeding from the root canals. After irrigation protocol with NaOCl 5.25%, NaCl, Octenidin, NaCl, CHX 0.2 % and NaCl 0.9% Ca(OH)2 paste was inserted as a temporary curative medication. A week later all four root canals were filled with Sealer® and gutta-percha points.

**Figure 2:**
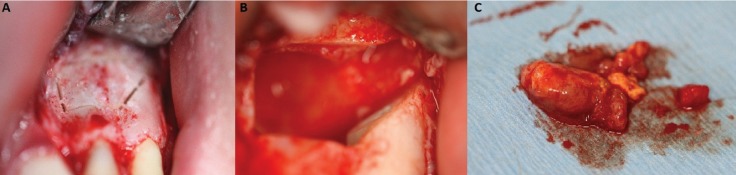
A) Piezocorticotomy design performed on area of teeth 22 and 23. B) Apicoectomy performed on teeth 22 and 23. C) Cystic material and apical portions of teeth 22 and 23 removed.

Surgery was performed under local anesthesia and its sequence is described below. First, teeth 18 and 28 were removed using conventional technique with mucosal incision and buccal osteotomy to expose the teeth. The two dental germs after being removed were cleaned with sterile NaCl 0.9%. Apical growth zone and rests of soft tissue were removed and both teeth were preserved in sterile NaCl 0.9%. On the sequence, two incisions and its mucosal flaps were elevated in the region of teeth 21-24 and 11-14. Piezosurgery was used for osteotomy of cortical bone and two cortical bone lids from the periapical areas were created and removed for preservation in NaCl 0.9% (Fig. 2A).

The resection of the root tips was performed and followed by removal of the cysts (Fig. 2B,C).

**Figure 3:**
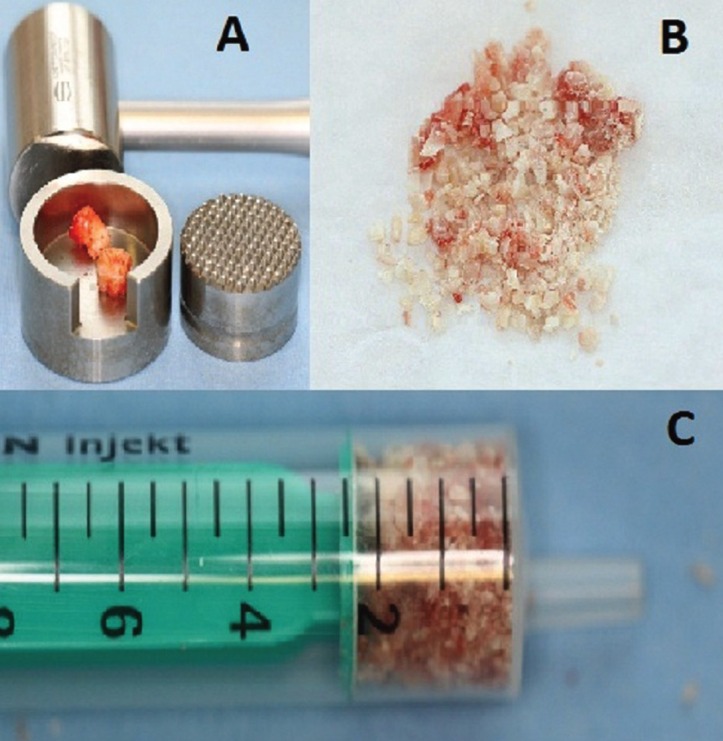
A) Grinder (crusher), mallet and teeth ready to be crushed.B) Different size of teeth milled, forming the autologous grafting material. C) Autologous bone substitute material ready to be inserted into residual bone defects.

Following our office developed “teeth crush technique” the two already extracted wisdom teeth were ground using bone mill and hammer (Fig. 3A-C). No chemical or physical conditioning was applied to these teeth. Several fragments, in different sizes, were formed with biggest ones up around to 2 mm.

Classical preparation of four retro cavities with piezo tips EN5R, EN5R-(Mectron, Germany) and insertion of retro fill with Ketac® (GC - Japan) was performed.

After bone defects curettage with curettes and rinsing with chlorhexidine solution the areas were filled with the particulate autogenous bone substitute grafting material.

**Figure 4:**
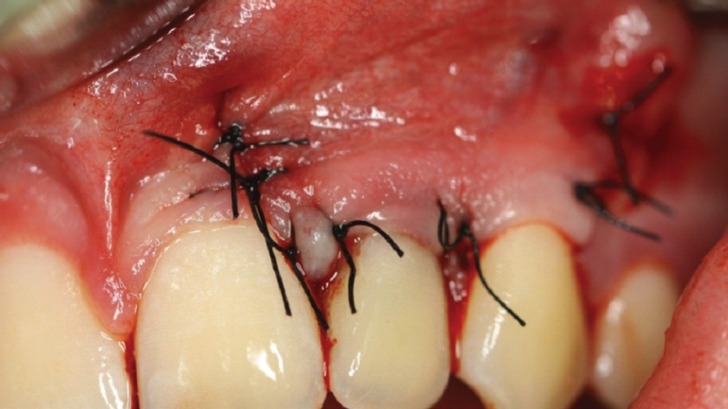
Final suture with Supramid® 3-0 (B. Braun, Germany), repositioning the mucosal flap in its initial position.

The two preserved bone lids were repositioned fitting perfectly to the primal osteotomy line. Atraumatic non-resorbable suture (Supramid® 3-0 - B. Braun, Germany) was used for the final soft tissue closure (Fig. 4). Additional support for teeth 22 was provided through wire splint for the first six months after the surgery.

The tentative diagnosis of radicular cysts was confirmed through histological examination.

**Figure 5:**
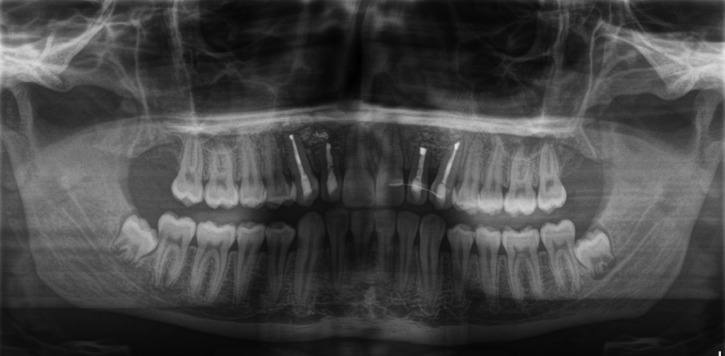
Panoramic X-ray after three months presenting the surgical site and the teeth which were involved in the procedures.

Patient´s postoperative Panoramic X-ray of six months after the surgery (Fig. 5) and clinical examination were corresponding to normal healing process.

## Discussion

Demineralized dentin (4) and Freeze Dried Dentin (5) have been more extensively researched and certainly they have a secure place as graft materials. But the processing of these materials takes place in a laboratory and the extracted teeth are cannot be immediately used. With the method the authors described in this paper the graft costs are minimal, is less time consuming, and the advantage of pulp cells preservation does not happen when grafts are processed in the laboratory. 

Clinical results advocate using teeth graft milled in special dentin grinder as alternative to autologous bone and Bio-Oss (5,6). The small size of this machine and simple protocol allow fast processing in the dental office but caries and foreign materials have to be removed with a bur. In addition to this preparations the teeth grinding must be followed by multiple-step chemical cleansings and bufferings. Because of that, the cost involved is not only the grinder but the solutions used as well. Certainly, this special dentin grinder eliminates the external processing but eliminate all of the pulp cells and their potential osteoinductive effect.

The authors describe a technique in which no investment is needed. A simple bone mill (bone crusher) and mallet which are commonly available in any dental office can be used. Autologous impacted intact teeth eliminate the need of chemical cleansing and/or buffering and caries removal. Also, dental pulp cells in this protocol are not removed nor chemically denatured which plays a very important role as far as bone formation is concerned.

Valdec *et al.* (8) used bone mill to particulate the autologous teeth. The graft preparation exclude chemical treatment similar to the wisdom tooth crush technique described in this research but the vital pulp tissue, enamel and cementum were completely removed. Those tissues were preserved in the protocol presented in this paper aiming to: 1) Increase the volume of graft per tooth, 2) Decrease the long time shrinkage of the material as we include inert and strong scaffold of dense hydroxyapatite crystallites (enamel), 3) Use the osteoinductive potential of the pulp cells.

The process followed by the authors on this case was similar to that used by Pohl *et al.* (10) and (11) which used intact impacted wisdom teeth. As on their series the last panoramic X-ray of the patient presented in this article revealed heterogenous material in the grafted sites but no radiolucent areas which represents bone formation involving the graft.

## Conclusions

Additional *in vivo* studies are required to confirm the predictability of wisdom teeth crush technique as a substitute graft material in bone defects and its osteointegration in grafted areas.

Given to its simple preparation protocol, potential benefits, low cost, and safety profile, the presented wisdom teeth crush technique can be taken into consideration by oral/maxillofacial surgeons as an autologous bone substitute augmentation/filling material.
